# Mind-body practice as a primer to maintain psychological health among pregnant women–YOGESTA–a randomized controlled trial

**DOI:** 10.3389/fpubh.2023.1201371

**Published:** 2023-09-12

**Authors:** Pooja Nadholta, Krishan Kumar, Pradip Kumar Saha, Vanita Suri, Amit Singh, Akshay Anand

**Affiliations:** ^1^Department of Neurology, Postgraduate Institute of Medical Education and Research, Chandigarh, India; ^2^Department of Psychiatry, Postgraduate Institute of Medical Education and Research, Chandigarh, India; ^3^Department of Obstetrics and Gynecology, Postgraduate Institute of Medical Education and Research, Chandigarh, India; ^4^Division of Yoga and Life Science, Swami Vivekananda Yoga Anusandhana Samsthana, Bengaluru, India; ^5^CCRYN-Collaborative Centre for Mind Body Intervention, PGIMER, Chandigarh, India

**Keywords:** pregnancy, Yoga, mind body intervention, neuropsychologial assessment, stress, prenatal Yoga, YOGESTA

## Abstract

**Objective:**

The objective of this study was to investigate the impact of Gestational Yoga-YOGESTA (Gestational Yoga), on the neuropsychology, quality of life, and personality of pregnant women.

**Design:**

Open label, randomized controlled trial, used allocation concealment to allocate the treatment.

**Setting:**

Department of Obstetrics and Gynecology and Neuroscience Research Lab, Department of Neurology, Post Graduate Institute of Medical Education and Research, Chandigarh, India.

**Participants:**

We recruited a total of 100 pregnant women visiting the Outpatient Department of Obstetrics and Gynecology. Participants were aged between 18 and 35 with uncomplicated pregnancies and they were randomly assigned to either the Yoga group (YG) or the usual care group (UCG). A total of 77 pregnant women completed both the pre- and post-survey, with 34 participants in the Yoga group and 43 in the Usual care group.

**Intervention:**

Pregnant women in their second and third trimesters were provided with a 16-week online Prenatal Yoga intervention. The intervention began after enrollment in the 2nd trimester, specifically between the 16th and 20th week, and was conducted 5 days a week until delivery, with an average intervention period of 47.18 ± 2.031 (mean ± SEM) days.

**Chief outcome measures:**

We measured Perceived stress, Depression, Anxiety, Stress, and quality of life by using standard questionnaires.

**Results:**

A total of 77 participants were included in the analysis, with 34 assigned to the Yoga group and 43 assigned to the control group. Most of the measured parameters demonstrated significant changes. The Yoga group exhibited a noteworthy decrease in perceived stress, depression, anxiety, and psychological stress, as well as an improvement in the psychological and environmental domains of QOL-BREF. Conversely, the control group demonstrated a significant increase in perceived stress, depression, anxiety, and psychological stress, along with a reduction in the physical, psychological, and social domains of QOL-BREF at the follow-up stage. Although the two groups were similar at baseline, the Yoga group showed substantial enhancements in perceived stress, depression, anxiety, physiological stress, and overall quality of life when compared to the control group at follow-up.

**Conclusion:**

The study’s findings indicate that stress, anxiety, and depression are more likely to occur as gestational age progresses during pregnancy. Nevertheless, practicing Prenatal Yoga can effectively manage these changes and enhance the quality of life for expectant mothers.

**Clinical trial registration**: Clinical Trials Registry-India, Identifier CTRI/2021/01/030827.

## Introduction

Pregnancy is marked by continuous physiological, metabolic, and mental challenges that can be difficult to adapt to. A woman’s neuropsychology and ability to manage these challenges effectively can play a crucial role in her adaptation to the physical and physiological demands of pregnancy. Stress, anxiety, and depression are common sources of distress during pregnancy that can have negative impacts on both maternal and fetal health (1). To ensure the smooth progression of pregnancy and fetal development, it is important to consider both biological and psychological factors such as anxiety, and physical and psychological stress, which may contribute to pregnancy-related complications (2). During the prenatal and postpartum periods, women experience significant changes in their psychological health, physiological function, and social interactions, all of which can have a significant impact on their lives. Stress, anxiety, and depression are the most common factors that affect neuropsychology during pregnancy and can contribute to various complications that arise during pregnancy (3). A study conducted in Sweden on a population of 1,734 pregnant women revealed that psychiatric disorders were prevalent in 14.1% of pregnant women, with 3.3% exhibiting signs of major depression, 6.9% showing signs of minor depression, and 6.6% displaying signs of anxiety during pregnancy (4). Studies have shown that women in late pregnancy experience poorer sleep quality, worse physical health, and higher rates of depression compared to women in early pregnancy (4). The physical and psychological changes brought on by pregnancy also impact the different aspects of quality of life (QOL). Additionally, poor quality of life has been linked to an increased risk of pregnancy complications such as premature birth and low birth weight. Therefore, healthcare providers should be aware of the psychological changes that occur during pregnancy and provide additional support to pregnant women (5). The effects of prolonged psychological stress on pregnant women include premature births, abnormal fetal development at birth, and attention disorders or reduced performance of new-born with regard to executive function in later life (6). It is important to manage stress levels in pregnant women and implement interventions that are designed to reduce psychological distress. However, it should be noted that psychopharmacological stress management may not be suitable for pregnant women due to concerns about the adverse effects of some commonly used pharmaceuticals (7). It is crucial to manage psychological distress during pregnancy for the well-being of both the mother and the fetus. Early detection and non-pharmacological management through techniques such as psychological counseling, stress management, lifestyle modifications (including light physical activity and a balanced diet), and social support can significantly improve the psychological health and quality of life of pregnant women (8). Incorporating non-pharmacological interventions, such as exercise and Yoga, has been shown to be effective in managing psychological problems during pregnancy. These interventions provide a proactive approach for pregnant women to promote their wellness during this critical time.

Existing research indicates that psychological distress can have a negative effect on the ability to participate in physical activity (9). Physical inactivity is a prevalent issue during pregnancy, with approximately 60% of pregnant women being sedentary. This is often due to a lack of awareness about the advantages of exercising during pregnancy, which may lead to hesitation among women. However, exercise during pregnancy is of utmost importance in terms of maternal and fetal health, as it effectively reduces common pregnancy issues such as depression, insomnia, anxiety, fatigue, and excessive weight gain in the mother (10, 11). With growing evidence and awareness of their benefits, exercise and Yoga have become more widely embraced and practiced by pregnant women (12). In general, exercise has a significant effect on the improvement of neuropsychology in adults as well as in older adults (13, 14). Given the limited feasibility of engaging in intense physical activities during pregnancy, gentle exercise practices such as Yoga may be a preferred option for pregnant women. Yoga can provide a combination of physical and mental wellness benefits that are well-suited to the needs of pregnant women.

Yoga is usually a mix of physical exercise, mental exercises, meditation, different types of deep breathing, stretching, and relaxation. The meditation component of Yoga promotes deep relaxation, which helps to calm the senses and improve the focus of the mind, thereby enhancing mental health. Practicing Yoga during pregnancy is known to promote a holistic connection between the mind, body, and fetus of expectant mothers (15). Previous research has shown promising results regarding the benefits of Yoga during pregnancy (16). Studies have demonstrated that practicing Yoga can reduce stress, anxiety, and depression, improve mood, and enhance overall well-being (17, 18). However, there is a need for further research to confirm and extend these findings.

Moreover, it is important to note that there is limited awareness about mental health issues during pregnancy, and routine screening for such problems is not common practice, thereby neglecting their significance as high-risk factors in pregnancy (19). However, it is crucial to acknowledge that psychological imbalances, including stress, anxiety, and psychosocial factors, have been associated with adverse outcomes such as preterm birth, low birth weight, and complications during both antepartum and intrapartum periods (19, 20). The generality ofthe need to carry out a counter-stress randomized controlled trial (RCT) in this region is imperative. It is worth mentioning that the labor room unpublished statistics of the hospital where our study was conducted revealed that out of a total of 5,202 deliveries in 2022, approximately 20.8% were preterm deliveries without a known cause. Psychological distress during pregnancy remains relatively understudied and often goes unnoticed as it is not routinely assessed during prenatal checkups. Hence the rationale of this study owes its origin to the need to investigate the incidence of psychological distress in pregnant women. We aimed to study the potential impact of Yoga on distress and its relationship to quality of life in healthy pregnant women. Our hypothesis is that regular practice of Yoga may enhance psychological resilience and lead to improved quality of life during pregnancy, thereby reducing the incidence of psychological distress. This study provides a rigorous and detailed investigation into the potential benefits of an online Yoga intervention for psychological distress and quality of life among uncomplicated pregnant women, and the randomized controlled design adds to the robustness of the study and the outcomes. By examining these variables in uncomplicated pregnant population, we hope to provide insights into the incidents of psychological distress during pregnancy, its progression throughout the period, and the potential role of Yoga as a preventive intervention for psychological distress during pregnancy. Further, this investigation might provide preliminary data about the incidents of psychological distress among pregnant women which can further be used to identify the mental health problem, its associated risks for maternal and fetal complications, and the need for psychological counseling during antenatal checkups.

Through our examination of these variables in women with uncomplicated pregnancies, our aim is to shed light on the occurrence and progression of psychological distress during pregnancy. Additionally, we seek to explore the potential role of Yoga as a preventive intervention for managing psychological distress during this critical period. Furthermore, this investigation may yield preliminary data regarding the incidence of psychological distress among pregnant women, which can contribute to identifying mental health issues, associated risks for maternal and fetal complications, and the need for psychological counseling during antenatal checkups.

## Materials and methods

### Study design and setting

The YOGESTA (Gestational Yoga) trial was carried out at the Post Graduate Institute of Medical Education and Research (PGIMER), Chandigarh, India after taking approval from the institutional ethics committee PGIMER.

This was an Open label, Parallel randomized controlled trial of online Yoga intervention among uncomplicated pregnant women. We recruited the participants in the 16th to 20th week of pregnancy and randomized them into Yoga and Usual care groups (UCG). Yoga group participants attended online morning Yoga classes from the time of recruitment until delivery, with daily attendance records and screenshots/videos of Yoga classes taken to ensure compliance with the intervention. UCG participants did not practice any Yoga or exercise during the study period. Assessment was done at two time points, baseline and follow-up. Baseline measurements were collected at the time of recruitment, i.e., between the 16th and the 20th week of pregnancy, and follow-up measurements were taken after 32 gestational weeks, using questionnaires to assess perceived stress (PSS); depression, anxiety, and stress (DASS); and quality of life (WHO-QOL BREF). The study design is both a between-subjects variable (comparing the Yoga group to the usual care group) and a within-subjects variable (comparing pre and post-intervention). The study was registered prospectively in the Clinical Trials Registry-India.

### Participants

From November 2021 to January 2023, pregnant women who visited or tele-consulted with the Department of Obstetrics and Gynecology at PGIMER were recruited for the study by the first author, based on defined inclusion criteria, which included uncomplicated normal pregnancy aged between 18 and 35 years in the 16th to 20th week with a BMI < 30 and no associated anomalies such as hypertension or gestational diabetes. Pregnancies with any associated comorbidities like hypertension, gestational diabetes mellitus, small cervical length, low-lying placenta, and high-risk pregnancies were excluded from the study. The sample size was estimated based on the mean and standard deviation from published research which had delivered Yoga interventions to the pregnant population, using the formula *n* = t2s2/r2m2 where *t* is the value of *t* statistic for 95% confidence, s is the standard deviation, r is the relative precision, taken as 0.05 in this case and m is the mean. The maximum sample size was then taken to ensure the estimation and testing of the hypothesis for the variable having maximum variance. The participants were invited by telephone or directly approached during antenatal clinic checkups, 100 agreed and provided written consent. The first author then randomly allocated the participants into the Yoga group (*n* = 50) and usual care group (*n* = 50) by using specific codes (A = Yoga group, B = Usual Care Group) concealed in sealed envelopes. The allocation ratio of randomization of the control to the Yoga group was 1:1. Whole process of recruitment and randomization was done by the first author. Blinding was not possible for this study due to the nature of the intervention, which involved participants actively engaging in Yoga sessions. As the participants were aware of their group assignment and actively participated in the Yoga intervention, it was not feasible to blind them or the researchers conducting the study to the treatment allocation. The protocol was reviewed and approved by the institutional ethical committee before the recruitment of participants. The final analysis included 77 participants (34 in the Yoga group and 43 in the usual care group) with a power level of 95% and an effect size of 0.5. The participants’ flow from baseline to follow-up is shown in Supplementary Table S1, as per the CONSORT flow chart. The participants’ age, BMI, and height did not differ between the two groups. The socioeconomic status [LIG- Low-Income group (5,000–16,000/month), MIG-1- Middle-Income group-1 (17,000–40,000/month), MIG-II- Middle Income group-II (41,000–85,000/month), HIG- High-Income group (85,000 and above)], demographics, and other parameters are reported as frequency and presented in Table 1.

**Table 1:** Characteristics and demographic details of participants.

Variables	Yoga group (*n* = 34)		Control group (*n* = 43)		
	*n*	%	*n*	%	*p*-value
**Age distribution**					
18–25	4	11.8	3	7.0	
26–30	18	52.9	23	53.5	0.752
31–35	12	35.3	17	39.5	
**Occupation**					
Housewives	26	76.5	26	60.5	0.151
Job	8	23.5	17	39.5	
**Education level**					
12th	7	20.6	11	23.6	0.248
Graduate	14	41.2	23	53.5	
Post graduate	13	38.2	9	20.9	
**Occupation of husband**					
Unemployed	0	0	0	0	
Employed	34	100	43	100	
**Smoke/alcohol**					
No	21	61.8	25	58.1	0.817
Yes	13	38.2	18	41.9	
**Parity distribution**					
Nulliparous	30	88.2	31	72.1	0.098
Multiparous	4	11.8	12	27.9	
**History of miscarriage**					
Yes	5	14.7	4	9.3	0.497
No	29	85.3	39	90.7	
**Year of marriage**					
2010–2014	1	2.9	6	14.0	0.126
2015–2020	33	97.1	37	86.0	
**Diet**					
Vegetarian	23	67.6	27	62.8	0.810
Non-vegetarian	11	32.4	16	37.2	
**Religion**					
Hindu	27	79.4	41	95.3	0.159
Sikh	5	14.7	2	4.7	
Jaat	1	2.9	0	0	
Jain	1	2.9	0	0	
**Socio-economic status**					
LIG	5	14.7	–	–	0.336
MIG-I	10	29.4	14	32.6	
MIG-II	9	26.5	14	32.6	
HIG	10	29.4	15	34.9	
Average Age Mean ± SEM	29.31 ± 3.415		29.71 ± 3.00		0.116
Average Height Mean ± SEM	1.58 ± 0.3293		1.59 ± 0.5269		0.412
BMI Mean ± SEM (in 2nd trimester)	22.32 ± 0.435		22.32 ± 0.378		0.413
BMI Mean ± SEM (in 3rd trimester)	26.09 ± 0.519		25.85 ± 0.401		0.414

Characteristics and demographics of participants in Yoga group (*n* = 34) and Usual care group (*n* = 43). Descriptive statistics was done to derive the frequency of variables and chi-square was done to obtain significance level of differences. LIG, Low-income group; MIG, Middle-income group; HIG, High-income group; BMI, Basal metabolic Rate; SEM, Standard Error Mean. Significance was observed at *p* ≤ 0.05.

### Prenatal Yoga protocol

To cater to the changing physiological needs of pregnant women, our study aimed to implement gestational Yoga which we abbreviated as YOGESTA among uncomplicated pregnant women. YOGESTA consists of two distinct Yoga protocols for the 2nd and 3rd trimesters. The protocols incorporated a range of practices including stretching, breathing, relaxation, and meditation (details presented in Supplementary Table S2). The Yoga protocol was adapted from the book “Yoga for Pregnancy” by *HR Nagarathna*, with necessary modifications made by the Institute Ethical Committee, in consultation with an obstetrician. The modifications were made keeping in mind the safety and comfort of pregnant women, given the online nature of the intervention. The protocol was designed as 60 min for the second trimester and 40 min for the third trimester. The intervention was delivered by certified Yoga experts via the Google Meet interface 5 days a week from the time of recruitment until delivery. All the sessions were delivered online by instructors who observed and corrected the postures of individual participants. Daily attendance was recorded for every participant throughout the period of intervention. Participants in the Yoga group attended Yoga sessions for an average of 47.18 ± 2.03 (Mean ± SEM) days. The UCG participants were contacted via telephone follow-up to ensure that they were not involved in any physical activity except normal walking.

Briefly, the protocol comprised of asana (physical postures), pranayama (breathing practices), kriya (tratak), meditation, and relaxation practices.

### Instrumentation

Multiple scales were used in this research study, including the PSS-10, DASS-42, and WHO-QOL-26, to comprehensively assess psychological distress in pregnant women and evaluate the impact of Yoga on these parameters, ensuring a comprehensive and multi-dimensional assessment of psychological well-being and quality of life.

### Perceived stress scale

The Perceived Stress Scale (PSS-10) is a 10-item stress assessment instrument originally, scale was developed in *1983* by *Cohen*, *Kamarck*, *and Mermelstein* (21). (PSS-10) was used to measure the perceived stress levels among pregnant women. It is a widely recognized and validated scale that assesses how various situations and events impact an individual’s thoughts and feelings, providing valuable insights into the specific stressors experienced during pregnancy. The scale asks questions related to negative events like how often you have been upset because of something that happened unexpectedly, how you felt you were unable to control important things in your life, and how often your felt nervous and stressed. Some questions are related to positive events which are revered during scoring, these are how often you felt confident about your ability to handle personal problems, how often you were able to control irritation, and how often you felt on top of things. Reliability, Validity, and scalability of PSS-10 during pregnancy have already been established by previous studies (22, 23) which show PSS-10 to be an appropriate scale to measure the perceived stress among pregnant women. The PSS measures an individual’s perceived stress, with scores ranging from 0 to 40. A score of 0–13 is considered low, 14–26 is moderate, and 27–40 is high.

### Depression anxiety stress scale

The Depression Anxiety Stress Scale (DASS-42) is a self-report measure that assesses the severity of negative emotions. The scale consists of three subscales, namely depression, anxiety, and stress, which are measured separately. Scores on each subscale can range from normal to extremely severe, depending on the severity of the symptoms reported by the individual. The severity levels are categorized as normal, mild, moderate, severe, and extremely severe (24). The scale contains 14 questions related to dysphoric moods such as sadness and unworthiness, measuring depression; 14 questions related to panic attacks and fears, measuring Anxiety, and the remaining 14 questions relate to tensions and irritability, measuring stress. Validation of DASS reliability during pregnancy has been done by previous studies (25, 26). (DASS-42) was employed to evaluate the severity of negative emotions, including depression, anxiety, and stress. By utilizing this comprehensive scale, we were able to capture a broad spectrum of psychological distress symptoms commonly experienced by pregnant women.

### Who- quality of life- BREF scale

Quality of life was measured using the WHO-QOL-BREF questionnaire which contains 26 original items, among which 2 items measure overall perception of quality of life and 24 items examines 4 domains (D1- Physical, D2- Psychological, D3- Social, and D4- Environmental) (27). This is also a reliable scale to be used during pregnancy (28). This questionnaire depicts score from 0 to 100 and a higher score signifies better QOL. This questionnaire was used to assess the overall quality of life of pregnant women across multiple domains, including physical, psychological, social, and environmental aspects. This scale provides a holistic understanding of the impact of pregnancy on various aspects of life.

## Results

In total, 100 participants gave their consent to participate and were randomized into Yoga (*n* = 50) and control or usual care groups (*n* = 50). During the course of the study, in the Yoga group, 16 participants dropped out for various reasons. Five of these individuals were not willing to participate in the Yoga intervention, eight started but discontinued the intervention, one participant experienced a miscarriage, and two did not complete the required neuropsychological assessments. In the Usual care group, five discontinued the trial, and two experienced miscarriages. When accounting for miscarriages, the attrition rate was found to be 23%. However, if miscarriages are not included, the attrition rate for the study is 20%. Finally, 77 participants who completed the neuropsychological assessment at both time points were included in the data analysis including 34 and 43 in YG and UCG groups, respectively.

The two groups were found to be similar in terms of age, height, weight, BMI, occupation, education level, parity distribution, diet, socio-economic status, pregnancy method, and complications, indicating no significant differences between them in these demographic and lifestyle factors (Table 1).

All results are reported as Mean ± SEM and frequencies in %. Frequency percentage was reported as low, moderate, and high for PSS and normal, mild, moderate, severe, and extremely severe for DASS as per the standard questionnaire classification using descriptive statistics (Table 3). Within-group changes and between-group changes of Yoga and control groups are reported as Mean ± SEM in Table 2 along with the effect size of each parameter and considering *p* ≤ 0.05 as a significant change. The effect size was calculated by dividing the difference of mean value between two groups by pooled standard deviation and is reported as a value of Cohen’s d where *d* = 0.2 is small, *d* = 0.5 is medium and *d* = 0.8 is considered as large based on the benchmark suggested by Cohen (29).

**Table 2:** Neuropsychological scoring within and between two groups.

Scale	Study group	Baseline Mean (SEM)	Follow-up Mean (SEM)	Average change Mean (SEM)	Effect size baseline vs. follow-up	Baseline vs. follow-up (*p* value)	Yoga vs. control *at baseline ^#^at follow-up (*p* value)	95% confidence interval of the difference (* baseline # followup)	
								Lower	Upper
Perceived stress scale	YG	17.38 (0.941)	13.41 (0.943)	3.971 (0.963)	0.658	**0.0001**	0.623^*^	−1.796	2.979
	UCG	16.79 (0.763)	20.84 (0.927)	−4.047 (0.898)	−0.73	**0.0001**	**0.0001** ^**#**^	−10.091	−4.76
Depression	YG	8.71 (1.441)	4.41 (0.998)	4.294 (0.992)	0.57	**0.0001**	0.408^*^	−1.923	4.684
	UCG	7.33 (0.938)	10.35 (1.191)	−3.023 (1.204)	−0.43	**0.016**	**0.0001** ^**#**^	−9.14	−2.734
Anxiety	YG	9.09 (0.946)	6.35 (0.774)	2.735 (1.011)	0.38	**0.011**	0.191^*^	−0.837	4.13
	UCG	7.44 (0.818)	9.93 (1.044)	−2.488 (0.992)	−0.41	**0.016**	**0.010** ^**#**^	−6.29	−0.865
Stress	YG	12.38 (1.346)	7.35 (1.010)	5.029 (0.981)	0.7	**0.0001**	0.691^*^	−2.728	4.097
	UCG	11.70 (1.090)	16.93 (1.342)	−5.233 (1.339)	−0.66	**0.0001**	**0.0001** ^**#**^	−13.078	−6.076
Physical domain	YG	64.71 (2.188)	67.76 (2.006)	−3.059(2.634)	0.18	0.254	0.199^*^	−8.892	1.885
	UCG	68.21 (1.672)	59.28 (2.190)	8.930 (2.393)	0.71	**0.001**	**0.007** ^**#**^	2.425	14.546
Psychological domain	YG	64.76 (2.732)	74.68 (2.789)	−9.912 (2.787)	0.67	**0.001**	0.19^*^	−10.784	2.173
	UCG	69.07 (1.924)	62.07 (2.229)	7.000 (1.928)	0.51	**0.001**	**0.001** ^**#**^	5.584	19.63
Social domain	YG	78.00 (3.336)	76.32 (3.218)	1.676 (2.884)	0.07	0.565	0.79^*^	−7.955	10.42
	UCG	76.77 (3.140)	67.05 (3.269)	9.721 (2.403)	0.46	**0.002**	**0.050** ^**#**^	−0.005	18.559
Environmental domain	YG	66.15 (2.913)	75.18 (2.358)	−9.029 (2.640)	0.64	**0.002**	0.597^*^	−9.252	5.36
	UCG	68.09 (2.310)	63.91 (2.572)	4.186 (2.269)	0.26	0.072	**0.002** ^**#**^	4.151	18.388

Baseline and follow-up changes in neuropsychology were compared between the Yoga group (*n* = 34) and usual care group (*n* = 43) at baseline and at follow-up using independent *t*-test and with the group using paired *t*-test. Significance was observed at *p* ≤ 0.05.

**Table 3:** Descriptive of PSS and DASS as per the severity.

	Yoga group (*N* = 34)		Usual care group (*N* = 43)	
Variables	Baseline *N* (%)	Followup *N* (%)	Baseline *N* (%)	Followup *N* (%)
**PSS**				
Low	9 (26.5%)	19 (55.9%)	9 (20.9%)	5 (11.6%)
Moderate	24 (70.6%)	15 (44.1%)	34 (79.1%)	29 (67.4%)
High	1 (2.9%)	–	–	9 (20.9%)
**Depression**				
Normal	24 (70.6%)	31(91.2%)	31 (72.16%)	21 (48.8%)
Mild	4 (11.8%)	1 (2.9%)	6 (14.0%)	10 (23.3%)
Moderate	4 (11.8%)	1 (2.9%)	5(11.6%)	9 (20.9%)
Severe	–	–	–	2 (4.7%)
Extremely severe	2 (5.9%)	1 (2.9%)	1 (2.9%)	1 (2.3%)
**Anxiety**				
Normal	16 (47.1%)	24 (70.6%)	25 (58.1%)	21 (48.8%)
Mild	3 (8.8%)	3 (8.8%)	2 (4.7%)	5 (11.6%)
Moderate	10 (29.4%)	5 (14.7%)	12 (27.9%)	6 (14.0%)
Severe	4 (11.8%)	1 (2.9%)	4 (9.3%)	5 (11.6%)
Extremely Severe	1 (2.9%)	1 (2.9%)	–	6 (14.0%)
**Stress**				
Normal	24 (70.6%)	30 (88.2%)	35 (81.4%)	18 (41.9%)
Mild	4 (11.8%)	2 (5.9%)	7 (16.3%)	4 (9.3%)
Moderate	2 (5.9%)	2 (5.9%)	1 (2.3%)	12 (27.9%)
Severe	3 (8.8%)	–	–	9 (20.9%)
Extremely severe	1 (2.9%)	–	–	–

Percentage of PSS (perceived stress scale) and DASS (depression anxiety stress scale) as per the severity at baseline and at follow-up in Yoga group (*n* = 34) and usual care group (*n* = 43).

### Perceived stress

When the average scores were compared, both groups reported the same level of stress at baseline (UCG: 16.79 ± 0.763, Yoga: 17.38 ± 0.943) without any significant differences (*p* = 0.623). However, within-group analysis after follow-up showed a significant increase in perceived stress from 16.79 ± 0.763 to 20.84 ± 0.927 (*p* = 0.000) in the UCG (average change: −4.047) and a significant decrease in perceived stress from 17.38 ± 0.943 to 13.41 ± 0.943 (*p* = 0.000) in the YG (average change: 3.971).

Between-group analysis at follow-up demonstrated a highly significant reduction in perceived stress in the Yoga group, with an average change of −7.425 (*p* = 0.000) compared to the UCG.

At baseline, the UCG reported a low level of perceived stress at 20.9%, while the remaining 79.1% reported a moderate level. These levels changed at follow-up, with only 11.6% reporting low stress, 67.4% reporting moderate stress, and 20.9% reporting high stress. However, the YG reported 26.5% low stress, 70.6% moderate stress, and 2.9% high stress at baseline, which changed to 55.9% low stress and 44.1% moderate stress after the intervention.

### Depression, anxiety, and stress

At baseline average score of the two groups did not differ for depression, anxiety, and stress. UCG reported a significant increase in depression from 7.33 ± 0.938 to 10.35 ± 1.191 (*p* = 0.016), anxiety from 7.44 ± 0.818 to 9.93 ± 1.044 (*p* = 0.016), and stress from 11.70 ± 1.090 to 16.93 ± 1.342 (*p* = 0.000) at follow-up. However the opposite trend was seen in YG with a significant decrease in depression from 8.71 ± 1.441 to 4.41 ± 0.998 (*p* = 0.000), anxiety from 9.09 ± 0.946 to 6.35 ± 0.774 (*p* = 0.011) and stress from 12.38 ± 1.346 to 7.35 ± 1.010 (*p* = 0.000) after intervention.

**In the UCG group,** at baseline, 72.16% were in the normal category of depression which decreased to 48.8% at follow-up. However, in mild, moderate, severe, and extremely severe categories percentages increased from 14.0, 11.6, 0, and 2.9%, to 23.3, 20.9, 4.7, and 2.3%, respectively. Similarly, the percentage of anxiety and stress in the normal range decreased from 58.1 and 81.4% to 48.8 and 41.9%, respectively. However, incidents of anxiety in the mild, moderate, severe, and extremely severe ranges increased from 4.7, 27.9, 9.3, and 0% to 11.6, 14.0, 11.6, and 14.0%, respectively. There were only 16.3% mild and 2.3% moderate incidents of stress while at follow-up only 9.3% were in the mild range, the moderate range increased to 27.9 and 20.9% were in the severe range.

**In the YG group,** the baseline there were 70.6% in the normal range of depression which increased to 91.2% after intervention, while the mild, moderate, and extremely severe range decreased to 2.9% only from 11.8 and 5.9%. Incidents in the normal range of anxiety and stress also increased after intervention from 47.1 and 70.6% to 70.7 and 88.2%, respectively. However, the mild and extremely severe ranges of anxiety remained the same at 8.8 and 2.9%, while the moderate and severe ranges decreased from 29.4 and 11.8% to 14.7 and 2.9%. Incidents of stress in the mild category decreased from 11.8 to 5.9%, remaining the same (i.e., 5.9%) in the moderate category and there were no incidents in the severe and extremely severe categories of stress after intervention which were 8.8 and 2.9% at baseline.

### Quality of life

Initially, no significant differences in the quality of life (QOL) were observed between the two groups. However, during the follow-up period, the group that received usual care (UCG) showed a significant decline in physical, psychological, and social domains, with scores decreasing from 68.21 ± 1.672 to 59.28 ± 2.190 (*p* = 0.001), 69.07 ± 1.924 to 62.07 ± 2.229 (*p* = 0.001), and 76.77 ± 3.140 to 67.5 ± 3.269 (*p* = 0.002), respectively, indicating a reduced quality of life with the progression of gestation. In contrast, the Yoga group reported a significant increase in psychological and environmental domains, with scores increasing from 64.76 ± 2.732 to 74.68 ± 2.789 (*p* = 0.001) and 66.15 ± 2.913 to 75.18 ± 2.358 (*p* = 0.002), respectively, indicating improved quality of life after Yoga.

Comparison of only follow-up data from both groups, the Yoga group demonstrated improved QOL, as evidenced by a significant average change of 8.486 (0.007), 12.607 (*p* = 0.001), 9.277 (0.050), and 11.269 (*p* = 0.002) in the physical, psychological, social and environmental domains, respectively, when compared to the UCG.

## Discussion

Our study underscores the impact of advancing gestational age on the neuropsychology of pregnant women, which can increase their vulnerability to symptoms of stress, anxiety, and depression. The findings emphasize the significance of prioritizing the psychological well-being of expectant mothers, particularly through Yoga, which can enhance their psychological resilience, as evidenced by the positive outcomes reported by the Yoga group. While the study findings suggest that Yoga can enhance psychological resilience in pregnant women, it is crucial to acknowledge the challenges associated with engaging a large population in such interventions. Despite offering the intervention at no cost and making it convenient to attend from home, with flexible scheduling options, a considerable number of eligible participants declined to participate and a few dropouts also occurred. This may indicate a need for healthcare providers to increase awareness among the pregnant population about the potential benefits of such interventions. Nonetheless, for those who are willing and able to engage in such programs, Yoga can provide significant benefits in terms of building psychological resilience and improving overall wellbeing. The intervention of prenatal Yoga demonstrated significant improvement in the psychological health of pregnant women, as reflected by reduced levels of perceived stress, depression, anxiety, and psychological stress. Furthermore, the intervention contributed to an overall enhancement of the quality of life. The effect size for the differences observed in the Yoga group was moderate to large, with a Cohen’s d value of 0.5 or greater. These findings suggest that the prenatal Yoga intervention had a substantial impact on the psychological well-being of pregnant women. In this study, we used a prenatal Yoga protocol that incorporated safe and gentle practices such as stretching, mild bending, meditation, breathing exercises, and relaxation techniques. These practices are considered to be beneficial to pregnant women and have been widely used for the general maintenance of their health. However, scientific evidence to support these claims has been limited until now. This study aimed to bridge this gap by designing a Yoga intervention that specifically focused on improving the attention and mental wellness of pregnant women. The results of our study suggest that this approach was effective in improving the psychological resilience and mental well-being of pregnant women, thereby highlighting the potential benefits of non-pharmacological interventions for this population. It should be noted that the practices used in the Yoga protocol were safe and adapted to the unique needs of pregnant women. Our study findings are in line with a previous study conducted by Abbas Rakhshani et al., which also reported improvement in psychological, social, and environmental domains of quality of life (QOL) through integrated Yoga practice during pregnancy (30). Our research further reinforces these findings by highlighting the positive impact of prenatal Yoga on the psychological and environmental domains of QOL, indicating a better outlook of pregnant women toward pregnancy after practicing Yoga. On the other hand, the usual care group reported a decrease in QOL domains of physical, psychological, and social health, indicating that physiological and psychological changes during pregnancy may have a greater impact on expectant mothers as pregnancy progresses, affecting their QOL and ultimately pregnancy outcomes. These changes can be better adapted by pregnant women if they integrate Yoga as a lifestyle during pregnancy, as our results show.

Kusuka et al. (17) have shown decreased salivary cortisol after each Yoga session in pregnant women. A negative indicator of mood such as anxiety, depression fatigue, and confusion, it was decreased after Yoga practice (17). We also find that perceived stress, anxiety, psychological stress, anxiety, and depression were significantly decreased in YG, whereas we found a significant increase shift in perceived stress and anxiety in UCG. Mindfulness Yoga was also effective in reducing depression symptoms and increasing maternal-fetal attachment and mindfulness among psychiatrically high-risk women (31), in addition to those in healthy pregnancies.

According to a study conducted by Field et al. (16), Yoga has advantages for pregnant women, including reducing stress, anxiety, and physical complaints throughout pregnancy, reducing discomfort, minimizing birthing pain, and speeding up the opening of the birth canal during birth. Yoga during pregnancy is thought to promote nervous system control and physiological system function (immunity, endocrine, neurotransmitter, and cardiovascular) as well as improve mental health to achieve a balance between the body and mind (16).

When compared to a waitlist control group, an RCT by Vieten et al. (32) evaluated a psychosocial mindfulness-based intervention administered in the second half of pregnancy reporting a reduction in anxiety and negative mood, indicating mindfulness-based interventions are a possible mental health approach to managing pregnancy stressors (32). Women who underwent mindfulness Yoga courses grew more comfortable with their position as mothers and valued their interactions with the fetus, according to a study by Muzik et al. (31), and they concluded that the supporting environment given in prenatal Yoga promotes the transition to safe motherhood. Prenatal Yoga encourages pregnant women to envisage and speak with their unborn child, which may explain why women who participated in Yoga courses had higher maternal-fetal attachment scores (31). Telephonic survey with the study participants after delivery revealed that participants of YG felt more connectedness with the fetus, more willpower, confidence to deal with stress and situations, relaxation, calmness, and positive attitude, incidents of which were much less in UCG as compared to YG.

Quality of life during pregnancy affects the pregnancy outcomes and impacts both the mother and the developing fetus. Depression and anxiety are independently associated with poor quality of life and vice versa which implies the need for healthcare professionals to give attention to the quality of life of women visiting prenatal clinics. Mild muscle relaxation exercise combined with music therapy has been shown to significantly improve quality of life parameters in pregnant women with low back pain (33). Likewise, mindfulness helps in bringing more coping strategies and management toward negative psychological emotions of mood and anxiety, and improves the quality of life which can ultimately improve pregnancy outcomes. Mindfulness Yoga intervention significantly reduced psychological distress symptoms and improve quality of life as reported by previous studies (34).

PSS measures the degree to which an individual perceives life to be unpredictable, uncontrollable, and overloaded for the previous month which can impact the psychological state of the individual. To predict specifically the role of Yoga in changing the perception toward their situation we individually compared the questions in the PSS scale, which depicts that after Yoga practice women were able to control their emotions in challenging situations, handle anger, and face difficulties with a more positive attitude, as suggested by the results. The present study specifically examines the effects of Yoga on both psychological distress and quality of life during pregnancy. By focusing on both outcomes, our study provides a comprehensive assessment of the potential benefits of Yoga during pregnancy. Additionally, it adds to the limited body of research on this topic, which will help inform healthcare providers and pregnant women about the potential benefits of incorporating Yoga into their prenatal care.

Based on the findings, Yoga may be used as a primer to reduce or prevent stress, anxiety, and depression-like symptoms, and improve QOL during pregnancy. One of the limitations of the study is the small sample size. We suggest that similar kinds of studies can be done using similar Yoga protocols to evaluate the impact on psychological parameters, quality of life, and other pregnancy outcomes on a bigger sample size and using molecular markers of the same parameters assessed in the study. To gain a deeper understanding of the impact of prenatal Yoga on pregnant women, future studies may consider exploring the molecular-level changes by analyzing Umbilical Cord Blood. Various biological components like cells, serum plasma from Yoga practitioners can be transplanted into animals to see the changes driven by Yoga at cellular and molecular level.

## Conclusion

Our study shows that the prenatal Yoga protocol used in this study was associated with reduced stress, anxiety, and depression among pregnant women. Therefore, this protocol can be used by pregnant women as a preventive as well as a therapeutic complementary measure for reducing pregnancy-induced stress, anxiety, and other psychological imbalances. Yoga appears to maintain good psychological health even in unhelpful emotional and mental states. Apart from psychological changes, our prenatal Yoga protocol was found to be helpful in alleviating overall quality of life by improving the quality of psychological and environmental health and balancing social and physical domains throughout the pregnancy.

### Statistical analysis

Within-group analysis was done using paired *t*-test while between-group analysis was done using an independent *t*-test. Statistical significance was considered a two-tailed value of *p* < 0.05. The frequency of the PSS and DAS scales was analyzed using descriptive statistics. All analyses were performed using IBM SPSS Statistics 21 software.

### Limitations of the study

Interpretation of our study outcome is made with caution given the number of limitations to our study design.

### Lack of blinding

Blinding of participants and researchers was not possible in this study due to the nature of the intervention. This may introduce bias in the assessment of outcomes and influence the results.

### Self-report measures

The study relied on self-report measures, which are subjective and can be influenced by participants’ interpretation and response bias, which may potentially affect the validity of the results.

### Sample characteristics

The study included only uncomplicated pregnant women aged between 18 and 35 years with a BMI < 30 and no associated anomalies. These narrow inclusion criteria may limit the generalizability of the findings to a broader population of pregnant women with different characteristics or complications.

### Compliance and adherence

The study aimed to ensure compliance with the intervention through daily attendance records and submission of screenshots/videos of Yoga classes. However, the accuracy and completeness of self-reported compliance data may be influenced by participants’ motivation, memory recall, and social desirability bias.

### Online intervention

The Yoga intervention was delivered online via Google Meet, which may have limitations compared to in-person classes. Factors such as internet connectivity, video and audio quality, and participants’ familiarity with technology could impact the effectiveness and engagement with the intervention.

## Strengths of the study

### Randomized controlled trial design

The study utilized a randomized controlled trial design, which is considered reliable for evaluating the effectiveness of interventions. Random allocation of participants into the Yoga group and usual care group helps minimize selection bias and increases the internal validity of the study.

### Prospective registration

The study was prospectively registered in the Clinical Trials Registry-India, which promotes transparency and helps prevent selective reporting of outcomes. This enhances the credibility and reliability of the study findings.

### Well-defined protocols

The study implemented two different Yoga protocols for the second and third trimesters of pregnancy, adapted from established sources and reviewed by an obstetrician and an institutional ethical committee. This standardized approach ensures consistency in the intervention delivery and allows for the reproducibility of the study in future research.

## Data availability statement

The raw data supporting the conclusions of this article will be made available by the authors, without undue reservation.

## Ethics statement

The studies involving human participants were reviewed and approved by Institutional Ethical Committee-Post Graduate Institute of Medical Education and Research (PGIMER). The patients/participants provided their written informed consent to participate in this study.

## Author contributions

AA conceptualized the whole study, edited the manuscript, and provided resources to complete the study. PN collected and sorted data, wrote the manuscript, and analyzed data. KK guided in doing neuropsychology of participants and reviewed and validated data and manuscripts. PS and VS provided the participants and assisted in the recruitment of participants. All authors contributed to the article and approved the submitted version.

## Conflict of interest

The authors declare that the research was conducted in the absence of any commercial or financial relationships that could be construed as a potential conflict of interest.

## Publisher’s note

All claims expressed in this article are solely those of the authors and do not necessarily represent those of their affiliated organizations, or those of the publisher, the editors and the reviewers. Any product that may be evaluated in this article, or claim that may be made by its manufacturer, is not guaranteed or endorsed by the publisher.
