# Supplementary material for: Mind-body practice as a primer to maintain psychological health among pregnant women–YOGESTA–a randomized controlled trial
Source: Front Public Health. 2023 Sep 12;11:1201371. doi: 10.3389/fpubh.2023.1201371 (PMC10520697; doi:10.3389/fpubh.2023.1201371)
Supplement: Supplementary file 1 [file Data_Sheet_1.PDF]

DASS

Name: *Date:*

Please read each statement and circle a number 0, 1, 2 or 3 which indicates how much the statement applied to you *over the past week*. There are no right or wrong answers. Do not spend too much time on any statement.

The rating scale is as follows:

0 Did not apply to me at all
1 Applied to me to some degree, or some of the time
2 Applied to me to a considerable degree, or a good part of time
3 Applied to me very much, or most of the time

1	I found myself getting upset by quite trivial things	0	1	2	3
2	I was aware of dryness of my mouth	0	1	2	3
3	I couldn't seem to experience any positive feeling at all	0	1	2	3
4	I experienced breathing difficulty (eg, excessively rapid breathing, breathlessness in the absence of physical exertion)	0	1	2	3
5	I just couldn't seem to get going	0	1	2	3
6	I tended to over-react to situations	0	1	2	3
7	I had a feeling of shakiness (eg, legs going to give way)	0	1	2	3
8	I found it difficult to relax	0	1	2	3
9	I found myself in situations that made me so anxious I was most relieved when they ended	0	1	2	3
10	I felt that I had nothing to look forward to	0	1	2	3
11	I found myself getting upset rather easily	0	1	2	3
12	I felt that I was using a lot of nervous energy	0	1	2	3
13	I felt sad and depressed	0	1	2	3
14	I found myself getting impatient when I was delayed in any way (eg, lifts, traffic lights, being kept waiting)	0	1	2	3
15	I had a feeling of faintness	0	1	2	3
16	I felt that I had lost interest in just about everything	0	1	2	3
17	I felt I wasn't worth much as a person	0	1	2	3
18	I felt that I was rather touchy	0	1	2	3
19	I perspired noticeably (eg, hands sweaty) in the absence of high temperatures or physical exertion	0	1	2	3
20	I felt scared without any good reason	0	1	2	3
21	I felt that life wasn't worthwhile	0	1	2	3

Please turn the page ☞

Reminder of rating scale:

0 Did not apply to me at all
1 Applied to me to some degree, or some of the time
2 Applied to me to a considerable degree, or a good part of time
3 Applied to me very much, or most of the time

22	I found it hard to wind down	0	1	2	3
23	I had difficulty in swallowing	0	1	2	3
24	I couldn't seem to get any enjoyment out of the things I did	0	1	2	3
25	I was aware of the action of my heart in the absence of physical exertion (eg, sense of heart rate increase, heart missing a beat)	0	1	2	3
26	I felt down-hearted and blue	0	1	2	3
27	I found that I was very irritable	0	1	2	3
28	I felt I was close to panic	0	1	2	3
29	I found it hard to calm down after something upset me	0	1	2	3
30	I feared that I would be "thrown" by some trivial but unfamiliar task	0	1	2	3
31	I was unable to become enthusiastic about anything	0	1	2	3
32	I found it difficult to tolerate interruptions to what I was doing	0	1	2	3
33	I was in a state of nervous tension	0	1	2	3
34	I felt I was pretty worthless	0	1	2	3
35	I was intolerant of anything that kept me from getting on with what I was doing	0	1	2	3
36	I felt terrified	0	1	2	3
37	I could see nothing in the future to be hopeful about	0	1	2	3
38	I felt that life was meaningless	0	1	2	3
39	I found myself getting agitated	0	1	2	3
40	I was worried about situations in which I might panic and make a fool of myself	0	1	2	3
41	I experienced trembling (eg, in the hands)	0	1	2	3
42	I found it difficult to work up the initiative to do things	0	1	2	3
