# Supplementary material for: Mind-body practice as a primer to maintain psychological health among pregnant women–YOGESTA–a randomized controlled trial
Source: Front Public Health. 2023 Sep 12;11:1201371. doi: 10.3389/fpubh.2023.1201371 (PMC10520697; doi:10.3389/fpubh.2023.1201371)
Supplement: Supplementary file 2 [file Data_Sheet_2.PDF]

Perceived Stress Scale

A more precise measure of personal stress can be determined by using a variety of instruments that have been designed to help measure individual stress levels. The first of these is called the **Perceived Stress Scale**.

The Perceived Stress Scale (PSS) is a classic stress assessment instrument. The tool, while originally developed in 1983, remains a popular choice for helping us understand how different situations affect our feelings and our perceived stress. The questions in this scale ask about your feelings and thoughts during the last month. In each case, you will be asked to indicate how often you felt or thought a certain way. Although some of the questions are similar, there are differences between them and you should treat each one as a separate question. The best approach is to answer fairly quickly. That is, don't try to count up the number of times you felt a particular way; rather indicate the alternative that seems like a reasonable estimate.

For each question choose from the following alternatives:
0 - never 1 - almost never 2 - sometimes 3 - fairly often 4 - very often

________ 1. In the last month, how often have you been upset because of something that happened unexpectedly?

________ 2. In the last month, how often have you felt that you were unable to control the important things in your life?

________ 3. In the last month, how often have you felt nervous and stressed?

________ 4. In the last month, how often have you felt confident about your ability to handle your personal problems?

________ 5. In the last month, how often have you felt that things were going your way?

________ 6. In the last month, how often have you found that you could not cope with all the things that you had to do?

________ 7. In the last month, how often have you been able to control irritations in your life?

________ 8. In the last month, how often have you felt that you were on top of things?

________ 9. In the last month, how often have you been angered because of things that happened that were outside of your control?

________ 10. In the last month, how often have you felt difficulties were piling up so high that you could not overcome them?

Figuring Your PSS Score

You can determine your PSS score by following these directions:

- First, reverse your scores for questions 4, 5, 7, and 8. On these 4 questions, change the scores like this:

  0 = 4, 1 = 3, 2 = 2, 3 = 1, 4 = 0.

- Now add up your scores for each item to get a total. **My total score is___________.**

- Individual scores on the PSS can range from 0 to 40 with higher scores indicating higher perceived stress.
  - ► Scores ranging from 0-13 would be considered low stress.
  - ► Scores ranging from 14-26 would be considered moderate stress.
  - ► Scores ranging from 27-40 would be considered high perceived stress.

The Perceived Stress Scale is interesting and important because your perception of what is happening in your life is most important. Consider the idea that two individuals could have the exact same events and experiences in their lives for the past month. Depending on their perception, total score could put one of those individuals in the low stress category and the total score could put the second person in the high stress category.

***Disclaimer**: The scores on the following self-assessment do not reflect any particular diagnosis or course of treatment. They are meant as a tool to help assess your level of stress. If you have any further concerns about your current well being, you may contact EAP and talk confidentially to one of our specialists.*
